# Airway Inflammation and Illness Severity in Response to Experimental Rhinovirus Infection in Asthma

**DOI:** 10.1378/chest.13-1567

**Published:** 2014-01-23

**Authors:** Jie Zhu, Simon D. Message, Yusheng Qiu, Patrick Mallia, Tatiana Kebadze, Marco Contoli, Christine K. Ward, Elliot S. Barnathan, Mary Ann Mascelli, Onn M. Kon, Alberto Papi, Luminita A. Stanciu, Peter K. Jeffery, Sebastian L. Johnston

**Affiliations:** From the Department of Respiratory Medicine (Drs Zhu, Message, Mallia, Kebadze, Contoli, and Stanciu and Prof Johnston), National Heart and Lung Institute, Medical Research Council, and Asthma UK Centre in Allergic Mechanisms of Asthma and Centre for Respiratory Infection, and Lung Pathology Unit (Drs Zhu and Qiu and Prof Jeffery), National Heart and Lung Institute, Imperial College London, London, England; Imperial College Healthcare NHS Trust (Drs Message, Mallia, Contoli, and Kon and Prof Johnston), London, England; Research Centre on Asthma and COPD (Drs Contoli and Papi), University of Ferrara, Ferrara, Italy; and Centocor Inc (Drs Ward, Barnathan, and Mascelli), Malvern, PA.

## Abstract

**Background:**

The nature of bronchial mucosal inflammation and its physiologic and clinical significance in rhinovirus-induced asthma exacerbations is unclear. We investigated bronchial mucosal inflammatory response and its association with physiologic and clinical outcomes in an experimental model of rhinovirus-induced asthma exacerbations.

**Methods:**

We used immunohistochemistry methods to detect phenotypes of inflammatory cells infiltrating the bronchial mucosa before and after experimental rhinovirus infection in 10 subjects with asthma and 15 normal subjects.

**Results:**

Compared with baseline, rhinovirus infection significantly increased the number of epithelial (*P* = .005) and subepithelial (*P* = .017) neutrophils in subjects with asthma only and subepithelial CD68^+^ macrophages in both subjects with asthma (*P* = .009) and normal subjects (*P* = .018) but more so in those with asthma (*P* = .021). Numbers of CD45^+^, CD68^+^, and CD20^+^ cells; neutrophils; and eosinophils at day 4 postinfection were positively associated with virus load (*r* = 0.50-0.72, *P* = .016-0.03). At acute infection in subjects with asthma, CD4^+^ cells correlated with chest symptom scores (*r* = 0.69, *P* = .029), the fall in the 10% fall in FEV_1_ (PC_10_) correlated with neutrophils (*r* = −0.89, *P* = .029), the PC_10_ correlated inversely with CD4^+^ (*r* = −0.67, *P* = .023) and CD8^+^ cells (*r* = −0.65, *P* = .03), the 20% fall in FEV_1_ was inversely associated with CD20^+^ cells (*r* = −0.65, *P* = .03), and higher epithelial CD8^+^ cell counts were significantly associated with a greater maximum fall in FEV_1_ (*r* = −0.72, *P* = .03), whereas higher subepithelial mast cell counts were significantly associated with a lower maximum percent fall in peak expiratory flow (*r* = 0.8, *P* = .024).

**Conclusions:**

In subjects with asthma, rhinovirus infection induces bronchial mucosal neutrophilia and more severe monocyte/macrophage infiltration than in normal subjects. Airway neutrophils, eosinophils, and T and B lymphocytes during infection are related to virus load and physiologic and clinical severity, whereas mast cells are related to greater lung function.

Rhinoviruses (RVs) are the major cause of acute exacerbations of asthma.^1‐3^ Human experimental RV infection in volunteers with mild asthma is associated with augmented physiologic and inflammatory responses to allergen challenge,^4,5^ reductions in peak expiratory flow (PEF)^6^ and FEV_1_,^7^ and increases in bronchial reactivity.^8^ In agreement, our own previously reported study showed that experimental RV16 infection in asthma induced significant increases in bronchial reactivity, lower respiratory tract symptoms, and lung function impairment and that significant changes in these outcomes did not occur in normal subjects.^9^

Regarding RV-induced airway inflammation, two previous studies of experimental RV infection reported increases in submucosal CD3^+^ lymphocytes and eosinophils in asthmatic and normal groups combined^10^ and in subjects with asthma alone.^11^ The increased number of mucosal CD3^+^ cells was accompanied by an increase in airway responsiveness and correlated positively with cold symptoms.^10,11^ However, it is unclear whether RV-induced bronchial mucosal cellular inflammatory responses differ between subjects with and without asthma.

In this article, we extend our previous work^9^ to compare the nature of the bronchial mucosa inflammatory response to experimental RV infection in subjects with asthma and in normal healthy subjects and explore its physiologic and clinical significance in asthma. We hypothesized that the bronchial mucosal cellular inflammatory response to RV infection will be exaggerated and of a distinctive predominant inflammatory cell phenotype in subjects with asthma compared with normal healthy subjects and that the inflammatory cells recruited to the bronchial mucosa will be associated with virus load and increased clinical symptoms, airways responsiveness, and airflow obstruction associated with an exacerbation.

## Materials and Methods

### Subjects

We extended our previously reported investigation of the same 10 subjects with atopic asthma and 15 subjects without atopic asthma (Table 1).^9^ These subjects were human RV16 neutralizing antibody seronegative. This study was conducted in accordance with the amended Declaration of Helsinki. All subjects gave written informed consent, and the study protocol was approved by the St. Mary’s NHS Trust Research Ethics Committee (99/BA/345). See e-Appendix 1 for other details.

**Table 1:** —Baseline Demographic Data

Subjects	Male (Female) Sex	Age, y	Serum IgE, U/mL	FEV_1_ % Predicted	Positive Skin Pick Test	Histamine PC_20_, mg/mL
Normal (n = 15)	8 (7)	27 ± 2.3	27 ± 8.0	104 ± 3.3	0	20 ± 2.5
With asthma (n = 10)	2 (8)	23 ± 1.4	241 ± 49.8^a^	106 ± 4.4	9	3 ± 0.7^a^

Data are presented as counts or mean ± SEM. PC_20_ = 20% fall in FEV_1_.

a *P* < .0001 vs normal (Student *t* test).

### Experimental Infection With Human RV16

Subjects were administered 10,000 tissue-culture infective dose 50% of RV16 on day 0 by nasal spray.^9^ See e-Appendix 1 for virologic confirmation of RV16 infection.

### Bronchoscopy and Clinical Data

Bronchial biopsy specimens were taken 14 days before infection (baseline), at day 4 (acute infection), and at 6 weeks postinfection (convalescence). For details about the physiologic and clinical data obtained, see e-Appendix 1.

### Immunohistochemistry

CD45^+^ pan-leukocyte and inflammatory cells, eosinophils, neutrophils, mast cells, and CD3^+^, CD4^+^, CD8^+^, and CD20^+^ cells were immunostained using previously described methods.^12^ For details on the immunostaining method, see e-Appendix 1.

### Quantification

The areas of epithelium and subepithelium in bronchial biopsy specimens were assessed using NIH Image, version 1.55 software (US National Institute of Mental Health). The inflammatory cells were counted using a light microscope. The data for cell counts were expressed as the number of positive cells per square millimeter of the subepithelium and per 0.1 mm^2^ epithelium. For details on the counting methods, see e-Appendix 1.

### Statistical Analysis

Within-group differences in cell counts between baseline and infection were assessed with Wilcoxon matched pairs test. Mann-Whitney *U* test was used to compare differences between groups. Spearman rank correlation was used to test for correlations between the numbers of phenotypes of inflammatory cells and physiologic and clinical data. *P* < .05 indicated significance. For further details on the statistical analyses, see e-Appendix 1.

## Results

**Figure 1.**
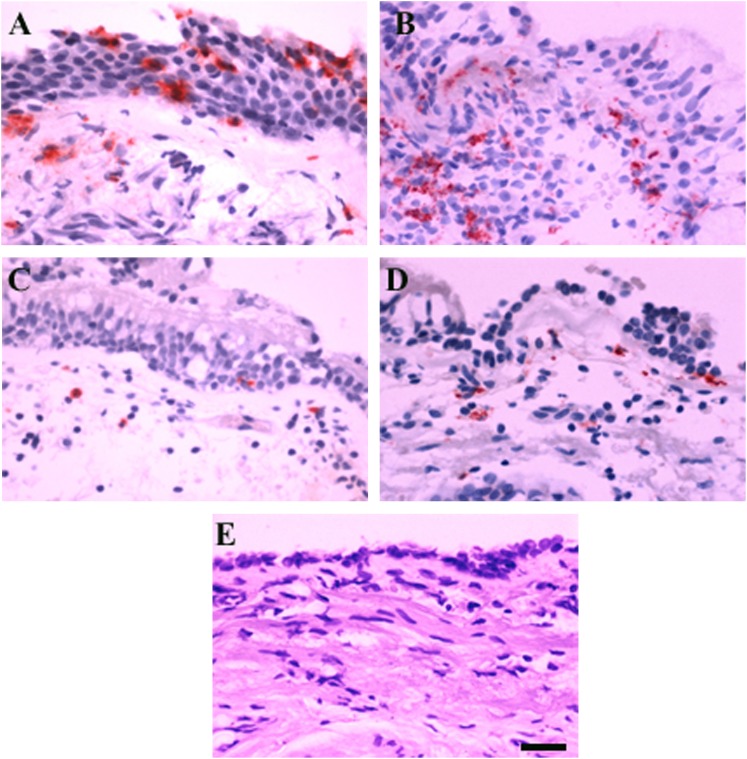
Immunohistochemistry-stained cells are seen as red fuchsin positivity. A and B, Subject with asthma at day 4 rhinovirus 16 infection demonstrating more elastase-positive neutrophils (A) and CD68^+^ monocytes/macrophages (B) in bronchial epithelium and subepithelium. C and D, Subject with asthma at baseline showing fewer neutrophils (C) and CD68^+^ cells (D). E, Negative control sample shows an absence of signal. Scale bar = 20 μm for all images.

Inflammatory cells were present in both the bronchial epithelial and the bronchial subepithelial compartments. Representative photographs are shown in Figures 1A‐E. Elastase-positive neutrophils (Fig 1A) and CD68^+^ monocytes/macrophages (Fig 1B) appeared to be more frequent in the bronchial mucosa of subjects with asthma during acute infection compared with baseline (Figs 1C, 1D). Application of irrelevant antibodies for the inflammatory cell markers was negative (Fig 1E).

### Absolute Counts of Inflammatory Cells

#### Total Leukocytes:

**Figure 2.**
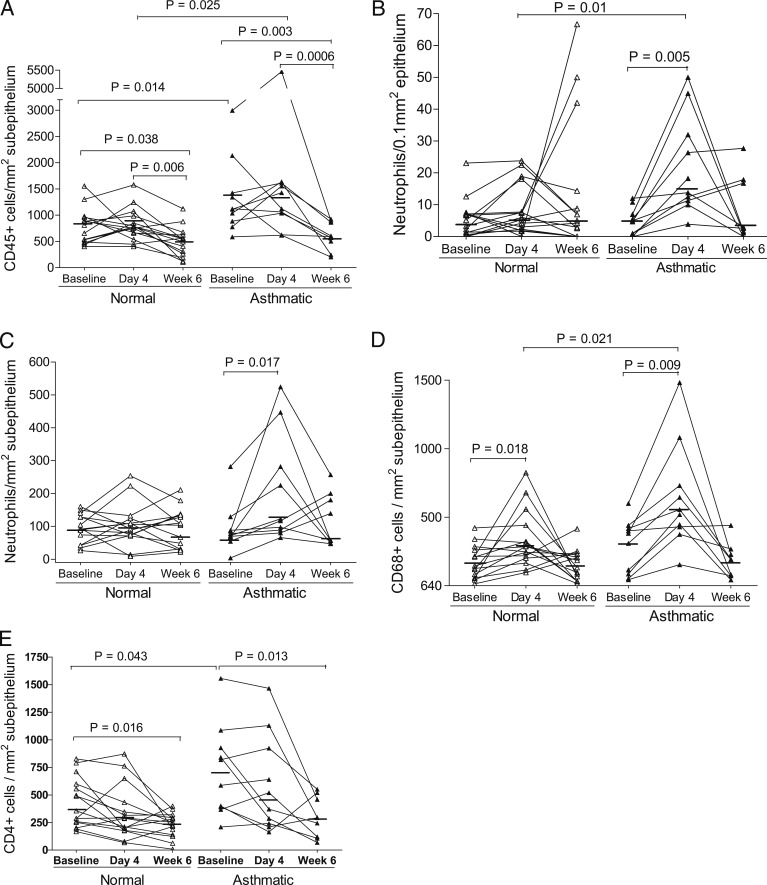
Cell counts in bronchial biopsy specimens of subjects with and without asthma at baseline and days 4 and 6 of rhinovirus 16 infection. A, CD45^+^ cells. B, Epithelial neutrophils. C, Subepithelial neutrophils. D, CD68^+^ cells. E, CD4^+^ cells. Data are presented as the number of positive cells per square millimeter of subepithelium or per 0.1 mm^2^ of epithelium. △ and ▲ show individual counts, and horizontal bars show median values (Wilcoxon matched pairs and Mann-Whitney *U* tests).

The number of subepithelial CD45^+^ cells in subjects with asthma was significantly greater at both baseline (*P* = .014) and day 4 infection (*P* = .025) (Fig 2A) than in normal subjects at the same time points. Compared with their respective baseline and day 4 values, the number of subepithelial CD45^+^ cells was significantly less at week 6 postinfection in both normal subjects (*P* = .038 and *P* = .006) and subjects with asthma (*P* = .003 and *P* = .0006) (Fig 2A). There were no significant differences in counts of epithelial CD45^+^ cells within and between groups.

#### Granulocytes:

Compared with baseline, the number of neutrophils in both epithelium (*P* = .005) (Fig 2B, Table 2) and subepithelium (*P* = .017) (Fig 2C) of the subjects with asthma but not of normal subjects were significantly increased at day 4. At this time point, there was a significantly greater number of epithelial neutrophils in the subjects with asthma (*P* = .01) (Fig 2B). EG2^+^ eosinophil counts were not significantly increased from baseline in either group after infection (Table 2). Compared with normal subjects, subjects with asthma had a significantly higher number of epithelial eosinophils at baseline (*P* = .011), day 4 (*P* = .046), and week 6 (*P* = .01) (Table 2) and showed trends toward a greater number of subepithelial eosinophils at baseline (*P* = .07) and day 4 (*P* = .09), which became statistically significant at week 6 (*P* = .03) (Table 2).

**Table 2:** —Counts of Phenotype of Inflammatory Cells Infiltrating Epithelial and Subepithelial Areas in Bronchial Mucosa of Subjects With and Without Asthma

	Asthma			Normal		
Group and Cells	Baseline	D 4	Wk 6	Baseline	D 4	Wk 6
Epithelium						
CD45^+^	126 (41-434)	164 (93-1,600)	111 (62-597)	109 (42-254)	152 (84-394)	113 (27-205)
Neutrophil elastase positive	4.9 (0-12)	16.1^a^ (4-50)	2.6 (0-28)	5.2 (0-23)	6 (0-24)	5 (6-67)
EG2^+^	8.4^b^ (0-160)	2.4^c^ (0-53)	1.2^d^ (0-4)	0 (0-8)	0 (0-9)	0 (0-2)
Tryptase positive	6 (0-15)	7^c^ (0-16)	11^d^ (3-24)	2 (0-41)	2 (0-7)	5 (0-13)
CD68^+^	14 (3-67)	28 (1-640)	24 (7-70)	22 (0-78)	30 (5-93)	36 (4-133)
CD3^+^	88 (20-145)	104 (44-452)	100^a^ (81-393)	97 (40-187)	99 (38-239)	125 (34-267)
CD4^+^	13 (2-38)	15 (3-65)	10 (2-55)	11 (0-26)	7 (1-35)	5 (0-27)
CD8^+^	64 (11-194)	80 (23-321)	77 (48-186)	79 (3-152)	70 (20-148)	76 (17-134)
CD20^+^	2 (0-7)	1 (0-4)	1 (0-3)	0 (0-12)	1 (0-15)	0 (0-2)
Subepithelium						
EG2^+^	84 (0-572)	55 (4-717)	26^d^ (1-133)	18 (0-214)	9 (0-202)	5 (0-50)
Tryptase positive	142 (78-252)	121 (8-285)	95 (16-243)	183^c,d^ (81-342)	92 (20-335)	58 (16-290)
CD3^+^	803 (292-1,280)	587 (159-2,127)	458 (241-1,406)	527 (225-1,091)	434 (91-1,261)	440 (99-1,373)
CD8^+^	712 (129-1,451)	536 (120-2,354)	457 (136-1,103)	407 (155-792)	418 (114-1,003)	306 (41-623)
CD20^+^	53 (17-322)	88 (15-351)	71 (10-275)	51 (0-386)	58 (4-379)	14 (0-98)

Data are presented as median (range) of positive cell counts per 0.1 mm epithelium and per square millimeter subepithelium.

a *P* = .005 and .023 vs asthma at baseline, respectively.

b *P* = .011 vs normal at baseline.

c *P* = .036, .046, and .049 vs normal on d 4, respectively.

d *P* = .006, .01, and .03 vs normal at wk 6, respectively.

#### Macrophages:

Compared with baseline, the number of subepithelial CD68^+^ cells in both normal subjects (*P* = .018) and subjects with asthma (*P* = .009) were significantly increased at day 4 of acute infection (Fig 2D). There was a significantly greater number of subepithelial CD68^+^ cells present in subjects with asthma than in normal subjects at day 4 (*P* = .021). No significant differences in counts of epithelial CD68^+^ cells were found within and between groups (Table 2).

#### Mast Cells:

There were no significant differences within the subjects with asthma in epithelial and subepithelial mast cell counts from baseline to day 4 or week 6 (Table 2). The number of subepithelial mast cells of normal subjects at baseline was unexpectedly higher than at day 4 (*P* = .036) and week 6 (*P* = .006) (Table 2). The number of epithelial tryptase-positive mast cells was significantly greater in subjects with asthma than in normal subjects at day 4 (*P* = .049) and week 6 (*P* = .01) (Table 2).

#### Lymphocytes:

In subjects with asthma only, the number of epithelial CD3^+^ cells increased at week 6 from baseline (*P* = .023) (Table 2). Unexpectedly, significant decreases in subepithelial CD4^+^ counts from baseline were seen at week 6 in both normal subjects (*P* = .016) and subjects with asthma (*P* = .013) (Fig 2E). Compared with normal subjects, subjects with asthma had a significantly greater number of subepithelial CD4^+^ cells at baseline (*P* = .043). There were no significant differences in epithelial CD4^+^, CD8^+^, or CD20^+^ cell counts and in subepithelial CD3^+^, CD8^+^, or CD20^+^ cell counts in either group after infection (Table 2).

### Changes in Numbers of Inflammatory Cells

**Figure 3.**
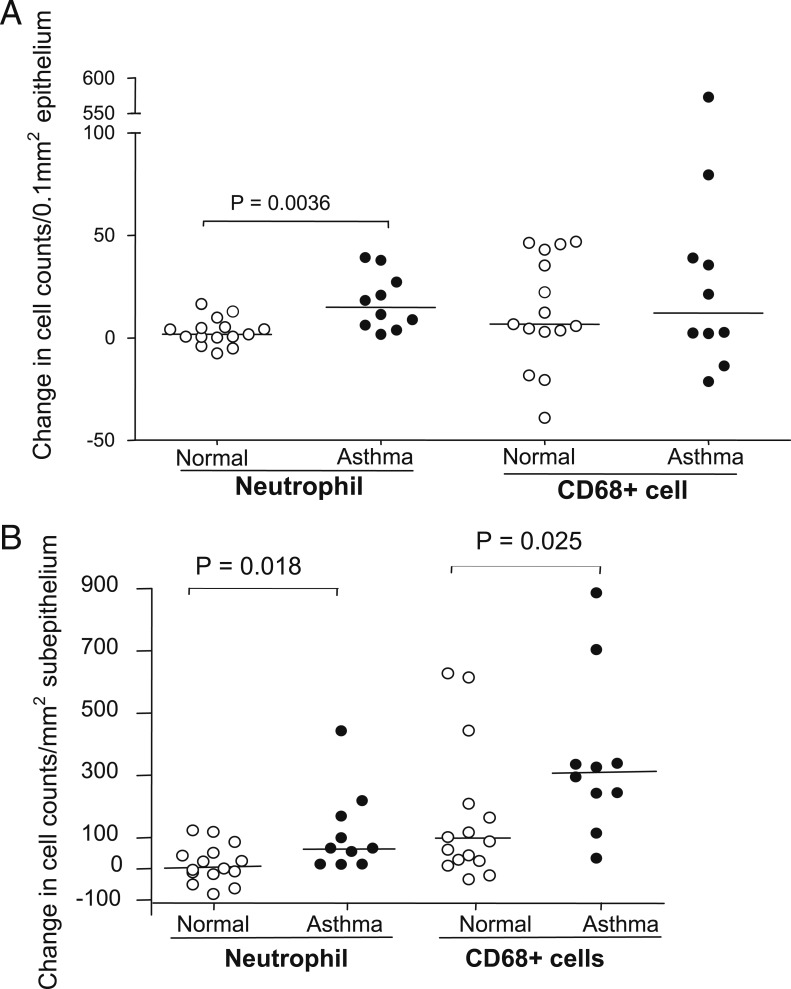
A and B, Changes in counts of epithelial (A) and subepithelial (B) neutrophils and CD68^+^ cells from baseline to d 4 postinfection in bronchial biopsy specimens from subjects with and without asthma. Data are presented as change in the number of positive cells per 0.1 mm^2^ of epithelium or per square millimeter of subepithelium. ○ and ● show individual changes in counts, and horizontal bars show median values (Mann-Whitney *U* test).

To investigate differences in inflammatory responses of subjects with and without asthma to RV infection, the magnitude of the changes of inflammatory cell counts from baseline to infection was compared. The changes in numbers of both epithelial (*P* = .0036) and subepithelial (*P* = .018) neutrophils from baseline to day 4 infection in subjects with asthma were significantly greater than those in normal subjects (Figs 3A, 3B). The changes in subepithelial CD68^+^ cell counts from baseline to day 4 infection in subjects with asthma were also significantly greater than those in normal subjects (*P* = .025) (Fig 3B). No significant differences with respect to changes in numbers of other inflammatory cell phenotypes were seen between groups from baseline to day 4 or week 6.

### Associations

#### Virus Load:

**Figure 4.**
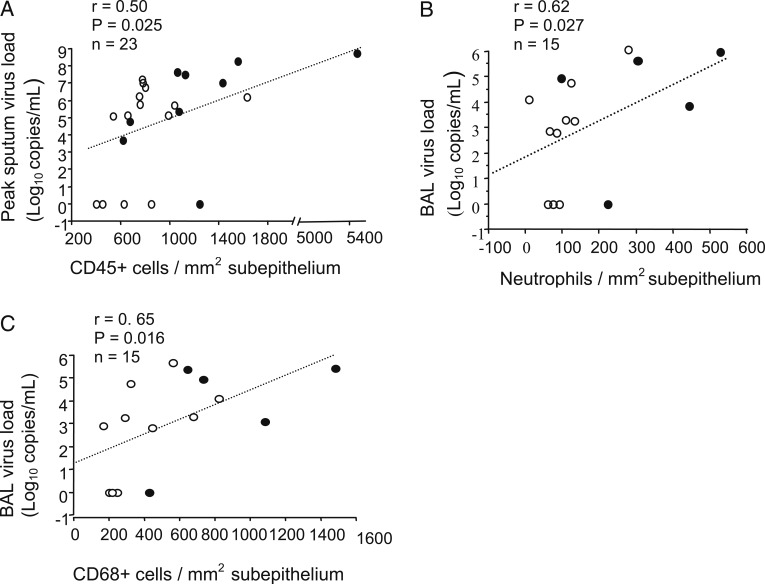
A-C, Correlations between the numbers of subepithelial CD45^+^ cells (A) at d 4 and peak sputum virus load at d 3 after infection and neutrophils (B) and CD68^+^ cells (C) at d 4 and BAL virus load at d 4 in all subjects with asthma (●) and normal subjects (○) (Spearman rank correlation).

In all subjects at day 4, the number of subepithelial CD45^+^ cells was associated with peak sputum virus load (*r* = 0.5, *P* = .025) (Fig 4A), and subepithelial neutrophils (*r* = 0.62, *P* = .027) (Fig 4B), EG2^+^ eosinophils (*r* = 0.64, *P* = .016), and CD68^+^ cells (*r* = 0.65, *P* = .016) (Fig 4C) correlated with BAL virus load.

#### Clinical Symptoms and Airway Hyperresponsiveness:

**Figure 5.**
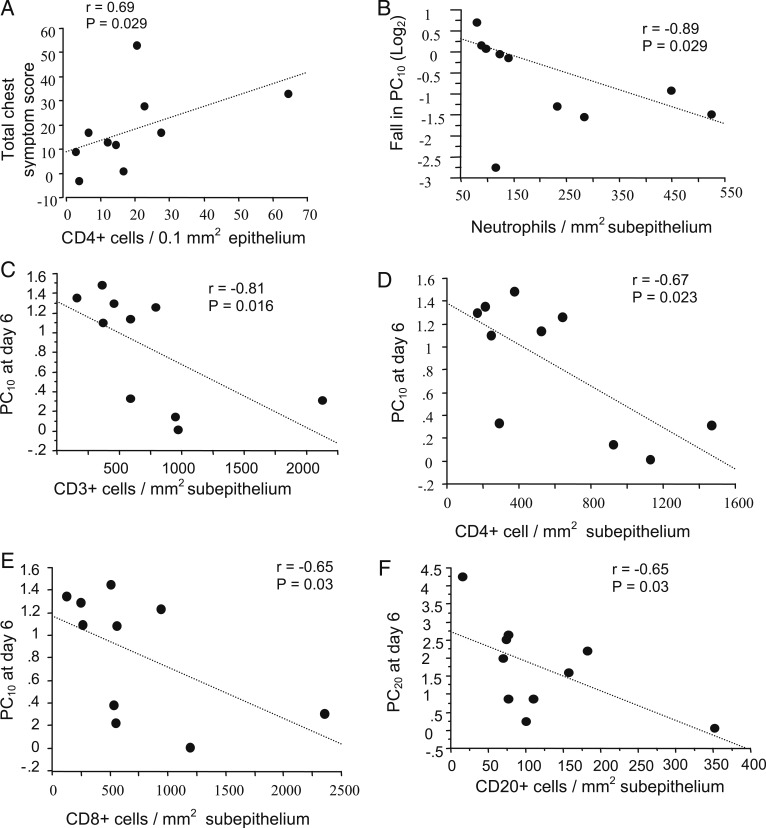
A-F, In subjects with asthma alone, correlations between total chest symptom score d 0 to 14 after the rhinovirus 16 infection period and counts of epithelial CD4^+^ cells (A), between fall in PC_10_ (log_2_) and counts of subepithelial neutrophils at d 4 (B), between PC_10_ at d 6 and counts of subepithelial CD3^+^ (C), CD4^+^ (D), and CD8^+^ (E) cells at d 4; and between PC_20_ at d 6 and counts of subepithelial CD20^+^ cells at d 4 (F) (Spearman rank correlation, n = 10 for all). PC_10_ = 10% fall in FEV_1_; PC_20_ = 20% fall in FEV_1_.

At day 4, epithelial CD4^+^ counts in subjects with asthma correlated with total chest symptom scores recorded during the 2-week postinfection period (*r* = 0.69, *P* = .029) (Fig 5A). Those with high subepithelial neutrophil counts had a significantly larger fall in the 10% fall in FEV_1_ (PC_10_) from baseline to day 6 postinfection (*r* = 0.89, *P* = .029) (Fig 5B). The PC_10_ at day 6 correlated inversely with subepithelial CD3^+^ (*r* = −0.81, *P* = .016) (Fig 5C), CD4^+^ (*r* = −0.67, *P* = .023) (Fig 5D), and CD8^+^ (*r* = −0.65, *P* = .03) (Fig 5E) cells, and the 20% fall in FEV_1_ at day 6 was inversely associated with subepithelial CD20^+^ cells (*r* = −0.65, *P* = .03) (Fig 5F).

#### Lung Function:

**Figure 6.**
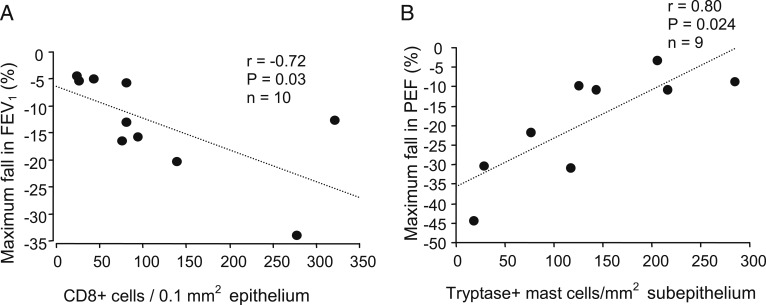
A, B, In subjects with asthma at d 4 after rhinovirus infection, correlations between maximum fall in FEV_1_ (%) and counts of epithelial CD8^+^ cells (A) and between maximum fall in PEF (%) and counts of subepithelial tryptase-positive mast cells (B) (Spearman rank correlation). PEF = peak expiratory flow.

At day 4, higher epithelial CD8^+^ cell counts in subjects with asthma were significantly associated with a greater maximum fall in FEV_1_ (percent fall from baseline) (*r* = −0.72, *P* = .03) (Fig 6A). Of interest, higher subepithelial mast cell counts were significantly associated with a lower maximum percent fall in PEF (*r* = 0.8, *P* = .024) (Fig 6B). In all subjects at day 4, higher epithelial (*r* = 0.64, *P* = .03) and subepithelial (*r* = −0.53, *P* = .01) CD4^+^ cell counts were significantly associated with a greater maximum fall in FEV_1_.

#### Serum IgE:

**Figure 7.**
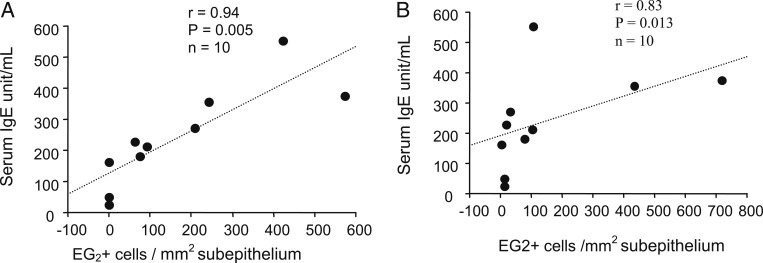
A, B, Correlations between serum IgE levels and counts of subepithelial EG2^+^ eosinophils in asthma at baseline (A) and d 4 after rhinovirus infection (B) (Spearman rank correlation).

The levels of serum IgE were significantly correlated with subepithelial EG2^+^ eosinophils in normal subjects at day 4 (*r* = 0.64, *P* = .017) and in subjects with asthma at both baseline (*r* = 0.94, *P* = .005) and day 4 (*r* = 0.83, *P* = .013) (Figs 7A, 7B). There were no significant associations between serum IgE level and other phenotypes of inflammatory cells.

## Discussion

We demonstrated that compared with normal subjects, subjects with asthma and RV infection had an exaggerated bronchial mucosal inflammatory response particularly characterized by increased numbers of neutrophils and CD68^+^ monocytes/macrophages. Significant correlations were found between CD45^+^ cells, neutrophils, and CD68^+^ cells and virus load and between neutrophils and T and B lymphocytes and airway hyperresponsiveness, chest symptoms, and reduced lung function, whereas mast cells were related to better lung function.

Previous experimental studies have shown that RV16 infection is associated with a significant increase in peripheral blood and BAL neutrophil counts in allergic asthma.^13^ In sputum, virus infection in asthma exacerbations has been associated with increased numbers of neutrophils and elevation of both neutrophil and eosinophil degranulation products, and cell lysis has been related to clinical severity.^14^ Increased numbers of neutrophils have been detected in the bronchial mucosa of subjects with severe asthma experiencing naturally occurring exacerbations.^15^ To our knowledge, however, no attempt has been made to determine whether RV infection alone can initiate bronchial mucosal neutrophilia, and its physiologic and clinical outcomes are unknown.

We reported in a previous publication on the clinical study from which the present biopsy specimens were collected increased numbers of BAL eosinophils during acute infection in the asthmatic group relative to the normal group, with similar, but nonsignificant trends for neutrophils.^9^ However, significant increases in BAL or induced sputum inflammatory cells from baseline to acute RV infection within the asthma group were not detected. Consistent with the BAL results, we found increased epithelial eosinophil and neutrophil counts in subjects with asthma vs normal subjects during acute RV infection. In addition, we demonstrated significant increases in the number of both epithelial and subepithelial neutrophils from baseline to acute infection in the asthma group, which were significantly higher than that in the normal group. We did not find increased numbers of neutrophils at week 6, which suggests that virus-induced airway neutrophilic inflammation in patients with mild asthma occurs only during the virus-induced acute asthma exacerbation and not during convalescence. Thus, in contrast to normal subjects, acute experimental RV16 infection selectively increases bronchial mucosal neutrophils in atopic asthma, and the higher numbers of neutrophils are related to the severity of infection and airway hyperresponsiveness. These data suggest that virus infection is one of the major etiologies inducing neutrophilic asthma. Because neutrophils play an important role in innate immune responses to clear virus, they can cause destruction of lung tissues through release of their proteases and other mediators.^16^ Therefore, the balance between the protective and pathologic effects of neutrophils in the course of virus-induced asthma exacerbations needs to be clarified when drug treatment to control neutrophilic inflammation^17^ is studied in the future.

Whereas we consider that asthma severity and treatment affect the magnitude of the inflammatory response to RV infection, the nature of the inflammation is more influenced by the time at which the biopsy specimens are taken in relation to the onset of RV infection. With respect to the latter, we believe that the results should not have been affected by the subjects with mild asthma studied who had not taken inhaled or oral corticosteroids.

The effect of RV infection on sputum and BAL CD68^+^ cells in subjects with and without asthma was not investigated in our previously reported study.^9^ Seymour et al^18^ reported an increase in the number of macrophages seen in bronchial biopsy specimens of normal subjects in response to experimental RV16 infection, but subjects with asthma were not investigated. In contrast, the present study shows that acute RV infection increases the number of subepithelial CD68^+^ cells of subjects with and without asthma. Notably, the changes in levels observed in the asthma group from baseline to acute infection were significantly greater than those in the normal group. Other work has suggested that impaired macrophage production of T-helper 1-related cytokines is strongly implicated in asthma exacerbation pathogenesis.^19,20^ Therefore, further investigation is needed to discern their role in asthma exacerbations induced by RV infection.

Airway mast cells are key in the immediate allergic response and are long associated with the pathogenesis of stable asthma.^21,22^ Using chloroacetate esterase to identify lysosomal granules in mast cells, Lozewicz et al^23^ found increased mast cell numbers in endobronchial biopsy specimens from subjects with atopic asthma compared with normal subjects. However, the numbers of tryptase-positive mast cells, tryptase-positive and chymase-negative mast cells, and tryptase- and chymase-positive mast cells in bronchial mucosa of subjects with symptomatic atopic asthma was not greater than that in normal subjects.^24,25^ We did not find a difference in the number of tryptase-positive mast cells in the bronchial mucosa between subject groups at baseline, which is consistent with these previous reports.^24,25^ Although there were no changes in numbers of epithelial mast cells within either group during RV infection, we detected a greater number of epithelial mast cells in asthma day 4 and week 6 compared with normal day 4 and week 6, implying that asthma susceptibility to immediate allergic response could be greater after RV infection. The numbers of subepithelial mast cells declined between baseline and acute infection and further declined between acute infection and convalescence in normal subjects. This finding appears to be inconsistent with an earlier study in which the number of tryptase-positive mast cells in bronchial submucosa declined between the infection and convalescent periods, but no initial increase was seen between baseline and infection.^26^ Of note, we detected in the present study that in subjects with asthma, those with the highest subepithelial mast cell counts had the lowest maximum percent falls in PEF during acute infection. In this regard, the findings support the results described previously in which the numbers of mast cells in severe asthma correlated with better rather than worse lung function, suggesting that recruitment of mast cells, particularly to the distal lung, may be protective for lung function in severe asthma.^27^

The biologic mechanism for a protective role remains unclear. Rauter et al^28^ notably demonstrated that β-tryptase, a major protease released during mast cell activation, cleaves IgE. They suggested that IgE cleavage by effector cell proteases is a natural mechanism for controlling allergic inflammation. The role of mast cells in the pathogenesis of virus-induced asthma exacerbations clearly merits further study.

We demonstrate for the first time, to our knowledge, in subjects with asthma that epithelial CD4^+^ cell counts at day 4 of infection correlates with total chest symptom scores, and the number of bronchial mucosal CD3^+^, CD4^+^, and CD8^+^ T and CD20^+^ B lymphocytes are associated with increased airway hyperresponsiveness. Additionally, the subjects with asthma with high epithelial CD8^+^ counts had significantly greater maximum falls in FEV_1_. The findings suggest the involvement of both T and B lymphocytes in the severity of asthma exacerbations induced by virus infection. The absence of increased counts of CD3^+^ and CD8^+^ T lymphocytes on day 4 of acute RV infection in subjects with asthma alone is not in keeping with increased subepithelial CD3^+^ and CD8^+^ cells at day 6 reported previously.^11^ This inconsistency may be due to differences in timing of the biopsies, as the present specimens were taken at day 4 vs day 6. The reductions in subepithelial CD4^+^ counts from baseline to convalescence may be due to a decrease in blood CD4^+^ cell counts during acute infection,^9^ which could be a result of less recruitment of CD4^+^ cells into the lung.

IgE plays a critical role in eosinophil recruitment into the airways after antigen provocation.^29^ Zambrano et al^30^ demonstrated that patients with mild asthma with high levels of IgE had significantly greater lower respiratory tract symptom scores during the initial 4 days of infection than those with low IgE levels. These patients also had higher total blood eosinophil counts at baseline. Building on these findings, we demonstrate that subjects with asthma with high levels of serum IgE at both baseline and day 4 have significantly greater numbers of submucosal EG2^+^ eosinophils. These findings imply that patients with atopic asthma with high levels of serum IgE could have more EG2^+^ eosinophils in their airway and experience more severe illness during acute RV infection. Anti-IgE therapy,^31,32^ therefore, may be useful for preventing exacerbations of asthma triggered by RV infection.

We acknowledge that an atopic nonasthma group was not included in the present study. Thus, the differences observed between the normal and asthma groups may have been partly due to changes attributable to atopy vs asthma. Previous work reported that compared with normal subjects without atopy, nasal biopsy specimens of subjects with atopy during experimental RV16 infection did not show increases in infiltration of inflammatory cells (including neutrophils).^33^ Another study showed no difference in changes of bronchial mucosa inflammatory cell counts between normal subjects and subjects with atopic rhinitis experiencing naturally occurring common cold virus infection.^34^ However, to answer this question directly, bronchial mucosa inflammatory responses to experimental RV16 infection in subjects with atopy but not asthma requires further investigation.

We also acknowledge that we have investigated relatively small numbers of subjects, albeit in some detail, and that a large number of statistical tests were carried out; thus, the study may have failed to detect some genuine observations due to limitations of statistical power. However, we consider its strengths to include the comprehensive and meticulous immunochemical approach and rigorous design of the human challenge model, permitting accurate sampling before and during infection of a uniform nature and known time of onset. Despite these caveats, novel data and interesting observations have emerged.

In conclusion, RV16 infection induced a more severe neutrophil and monocyte/macrophage-rich inflammatory response in the bronchial mucosa of subjects with asthma compared with normal subjects similarly infected. Numbers of mucosa neutrophils, monocytes/macrophages, EG2^+^ eosinophils, and CD4^+^, CD8^+^, and CD20^+^ cells in subjects with asthma during acute infection were related to virus load, increasing airway reactivity, and worsening of asthma symptoms and lung function, whereas tryptase-positive mast cells were related to better lung function. These data add to our understanding of the pattern of inflammatory response occurring during RV-induced experimental exacerbations of asthma and, as such, could have an impact on the design of future treatment modalities.

## Supplementary Material

Online Supplement

## Abbreviations

PC_10_: 10% fall in FEV_1_
PEF: peak expiratory flow
RV: rhinovirus

## References

[1] Nicholson KG Kent J Ireland DC Respiratory viruses and exacerbations of asthma in adults. BMJ. 1993;307(6910):982-986 8241910 10.1136/bmj.307.6910.982 PMC1679193

[2] Johnston SL Pattemore PK Sanderson G Community study of role of viral infections in exacerbations of asthma in 9-11 year old children. BMJ. 1995;310(6989):1225-1229 7767192 10.1136/bmj.310.6989.1225 PMC2549614

[3] Jackson DJ Sykes A Mallia P Johnston SL Asthma exacerbations: origin, effect, and prevention. J Allergy Clin Immunol. 2011;128(6):1165-1174 22133317 10.1016/j.jaci.2011.10.024 PMC7172902

[4] Lemanske RF Jr Dick EC Swenson CA Vrtis RF Busse WW Rhinovirus upper respiratory infection increases airway hyperreactivity and late asthmatic reactions. J Clin Invest. 1989;83(1):1-10 2536042 10.1172/JCI113843 PMC303635

[5] Calhoun WJ Dick EC Schwartz LB Busse WW A common cold virus, rhinovirus 16, potentiates airway inflammation after segmental antigen bronchoprovocation in allergic subjects. J Clin Invest. 1994;94(6):2200-2208 7989575 10.1172/JCI117581 PMC330045

[6] Bardin PG Fraenkel DJ Sanderson G van Schalkwyk EM Holgate ST Johnston SL Peak expiratory flow changes during experimental rhinovirus infection. Eur Respir J. 2000;16(5):980-985 11153603 10.1183/09031936.00.16598000

[7] Grünberg K Timmers MC de Klerk EP Dick EC Sterk PJ Experimental rhinovirus 16 infection causes variable airway obstruction in subjects with atopic asthma. Am J Respir Crit Care Med. 1999;160(4):1375-1380 10508832 10.1164/ajrccm.160.4.9810083

[8] Cheung D Dick EC Timmers MC de Klerk EP Spaan WJ Sterk PJ Rhinovirus inhalation causes long-lasting excessive airway narrowing in response to methacholine in asthmatic subjects in vivo. Am J Respir Crit Care Med. 1995;152(5):1490-1496 7582282 10.1164/ajrccm.152.5.7582282

[9] Message SD Laza-Stanca V Mallia P Rhinovirus-induced lower respiratory illness is increased in asthma and related to virus load and Th1/2 cytokine and IL-10 production. Proc Natl Acad Sci U S A. 2008;105(36):13562-13567 18768794 10.1073/pnas.0804181105 PMC2528869

[10] Fraenkel DJ Bardin PG Sanderson G Lampe F Johnston SL Holgate ST Lower airways inflammation during rhinovirus colds in normal and in asthmatic subjects. Am J Respir Crit Care Med. 1995;151(3 pt 1):879-886 7881686 10.1164/ajrccm/151.3_Pt_1.879

[11] Grünberg K Sharon RF Sont JK Rhinovirus-induced airway inflammation in asthma: effect of treatment with inhaled corticosteroids before and during experimental infection. Am J Respir Crit Care Med. 2001;164(10):1816-1822 11734429 10.1164/ajrccm.164.10.2102118

[12] O’Shaughnessy TC Ansari TW Barnes NC Jeffery PK Inflammation in bronchial biopsies of subjects with chronic bronchitis: inverse relationship of CD8+ T lymphocytes with FEV1. Am J Respir Crit Care Med. 1997;155(3):852-857 9117016 10.1164/ajrccm.155.3.9117016

[13] Jarjour NN Gern JE Kelly EA Swenson CA Dick CR Busse WW The effect of an experimental rhinovirus 16 infection on bronchial lavage neutrophils. J Allergy Clin Immunol. 2000;105(6pt 1):1169-1177 10856152 10.1067/mai.2000.106376

[14] Wark PA Johnston SL Moric I Simpson JL Hensley MJ Gibson PG Neutrophil degranulation and cell lysis is associated with clinical severity in virus-induced asthma. Eur Respir J. 2002;19(1):68-75 11852895 10.1183/09031936.02.00226302

[15] Qiu Y Zhu J Bandi V Guntupalli KK Jeffery PK Bronchial mucosal inflammation and upregulation of CXC chemoattractants and receptors in severe exacerbations of asthma. Thorax. 2007;62(6):475-482 17234659 10.1136/thx.2006.066670 PMC2117215

[16] Smith JA Neutrophils, host defense, and inflammation: a double-edged sword. J Leukoc Biol. 1994;56(6):672-686 7996043 10.1002/jlb.56.6.672

[17] Nagarkar DR Wang Q Shim J CXCR2 is required for neutrophilic airway inflammation and hyperresponsiveness in a mouse model of human rhinovirus infection. J Immunol. 2009;183(10):6698-6707 19864593 10.4049/jimmunol.0900298 PMC2952174

[18] Seymour ML Gilby N Bardin PG Rhinovirus infection increases 5-lipoxygenase and cyclooxygenase-2 in bronchial biopsy specimens from nonatopic subjects. J Infect Dis. 2002;185(4):540-544 11865407 10.1086/338570

[19] Contoli M Message SD Laza-Stanca V Role of deficient type III interferon-lambda production in asthma exacerbations. Nat Med. 2006;12(9):1023-1026 16906156 10.1038/nm1462

[20] Laza-Stanca V Message SD Edwards MR The role of IL-15 deficiency in the pathogenesis of virus-induced asthma exacerbations. PLoS Pathog. 2011;7(7):e1002114 21779162 10.1371/journal.ppat.1002114 PMC3136447

[21] Yamaya M Sasaki H Rhinovirus and asthma. Viral Immunol. 2003;16(2):99-109 12828863 10.1089/088282403322017857

[22] Galli SJ New concepts about the mast cell. N Engl J Med. 1993;328(4):257-265 8418407 10.1056/NEJM199301283280408

[23] Lozewicz S Gomez E Ferguson H Davies RJ Inflammatory cells in the airways in mild asthma. BMJ. 1988;297(6662):1515-1516 3147053 10.1136/bmj.297.6662.1515 PMC1835213

[24] Bradley BL Azzawi M Jacobson M Eosinophils, T-lymphocytes, mast cells, neutrophils, and macrophages in bronchial biopsy specimens from atopic subjects with asthma: comparison with biopsy specimens from atopic subjects without asthma and relationship to bronchial hyperresponsiveness. J Allergy Clin Immunol. 1991;88(4):661-674 1918731 10.1016/0091-6749(91)90160-p

[25] Djukanović R Wilson JW Britten KM Quantitation of mast cells and eosinophils in the bronchial mucosa of symptomatic atopic asthmatics and healthy control subjects using immunohistochemistry. Am Rev Respir Dis. 1990;142(4):863-871 2221594 10.1164/ajrccm/142.4.863

[26] Thomas LH Fraenkel DJ Bardin PG Johnston SL Holgate ST Warner JA Leukocyte responses to experimental infection with human rhinovirus. J Allergy Clin Immunol. 1994;94(6):1255-1262 7528234 10.1016/0091-6749(94)90340-9

[27] Balzar S Chu HW Strand M Wenzel S Relationship of small airway chymase-positive mast cells and lung function in severe asthma. Am J Respir Crit Care Med. 2005;171(5):431-439 15563633 10.1164/rccm.200407-949OC

[28] Rauter I Krauth MT Westritschnig K Mast cell-derived proteases control allergic inflammation through cleavage of IgE. J Allergy Clin Immunol. 2008;121(1):197-202 17904627 10.1016/j.jaci.2007.08.015

[29] Coyle AJ Wagner K Bertrand C Tsuyuki S Bews J Heusser C Central role of immunoglobulin (Ig) E in the induction of lung eosinophil infiltration and T helper 2 cell cytokine production: inhibition by a non-anaphylactogenic anti-IgE antibody. J Exp Med. 1996;183(4):1303-1310 8666888 10.1084/jem.183.4.1303 PMC2192518

[30] Zambrano JC Carper HT Rakes GP Experimental rhinovirus challenges in adults with mild asthma: response to infection in relation to IgE. J Allergy Clin Immunol. 2003;111(5):1008-1016 12743565 10.1067/mai.2003.1396

[31] D’Amato G Perticone M Bucchioni E Salzillo A D’Amato M Liccardi G Treating moderate-to-severe allergic asthma with anti-IgE monoclonal antibody (omalizumab). An update. Eur Ann Allergy Clin Immunol. 2010;42(4):135-140 21114196

[32] Busse WW Morgan WJ Gergen PJ Randomized trial of omalizumab (anti-IgE) for asthma in inner-city children. N Engl J Med. 2011;364(11):1005-1015 21410369 10.1056/NEJMoa1009705 PMC3093964

[33] Fraenkel DJ Bardin PG Sanderson G Lampe F Johnston SL Holgate ST Immunohistochemical analysis of nasal biopsies during rhinovirus experimental colds. Am J Respir Crit Care Med. 1994;150(4):1130-1136 7921447 10.1164/ajrccm.150.4.7921447

[34] Trigg CJ Nicholson KG Wang JH Bronchial inflammation and the common cold: a comparison of atopic and non-atopic individuals. Clin Exp Allergy. 1996;26(6):665-676 8809424 10.1111/j.1365-2222.1996.tb00593.x PMC7164830

