# Supplementary material for: Airway Inflammation and Illness Severity in Response to Experimental Rhinovirus Infection in Asthma
Source: Chest. 2014 Jan 23;145(6):1219–29. doi: 10.1378/chest.13-1567 (PMC4042510; doi:10.1378/chest.13-1567)
Supplement: Supplementary file 1 [file chest_145_6_1219_ds01.pdf]

CHEST Online Supplement

Airway Inflammation and Illness Severity in Response to Experimental Rhinovirus Infection in Asthma

Jie Zhu, MD, PhD; Simon D. Message, MD, PhD; Yusheng Qiu, MD, PhD; Patrick Mallia, MD, PhD; Tatiana Kebadze, MD; Marco Contoli, MD; Christine K. Ward, PhD; Elliot S. Barnathan, MD; Mary Ann Mascelli, PhD; Onn M. Kon, MD; Alberto Papi, MD, FCCP; Luminita A. Stanciu, MD, PhD; Peter K. Jeffery, PhD, DSc; and Sebastian L. Johnston, PhD

e-Appendix 1.

Subjects: we here extended our investigation of the same 25 non-smokers reported previously.[2] Those were human RV16 neutralizing antibody sero-negative and comprised of: 10 atopic asthmatics and 15 normal non atopic adults (Table 1).

Clinical and atopic status were defined by questionnaire, skin prick testing, serum IgE and lung function testing including PEF, FEV_1, forced vital capacity (FVC). [2] The normal healthy subjects were taking no medication. Asthmatics were prescribed inhaled short-acting β_2-agonists only. Subjects were free of common cold symptoms for at least 6 weeks prior to the study start.

Histamine challenge. Bronchial reactivity was measured by histamine challenge at baseline, on day 6 after infection and convalescence. For bronchodilator reversibility, sputum induction and histamine challenge subjects used a Vitalograph Dry Wedge Bellows Spirometer. Histamine challenge (maximum concentration 32 mg/ml) was performed according to guidelines by using the 2-min tidal breathing method.[1] The asthmatic group was required to have a PC20 < 8 mg/ml, and the normal group had > 8 mg/ml. The provocative concentration of histamine causing a 20% reduction in FEV1 (PC20) was

Online supplements are not copyedited prior to posting.

©2014 American College of Chest Physicians. Reproduction of this article is prohibited without written permission from the American College of Chest Physicians. See online for more details. DOI: 10.1378/chest.13-1567
CHEST 2014; 145(6):1219-1229

calculated,[1] and as eight normal subjects did not achieve a 20% reduction, a PC10 was also calculated for both groups.

Skin Prick Testing and Serum IgE. Atopy was determined by skin prick testing to common aeroallergens: six grass pollen mix; house dust mite; cat; dog; *Aspergillus fumigatus*; *Cladosporium herbarum*; *Alternaria alternata*; and birch, three tree and nettle pollen (ALK Abello). One wheal 3 mm greater than negative

control or total IgE > 110 units/ml was considered diagnostic of atopy.[2]

Virologic confirmation of RV16 infection

RV16 infection was confirmed by at least one of the following: positive standard or qPCR for RV or positive culture of RV16 from induced sputum obtained on days 3 and 7 and bronchoalveolar lavage (BAL) on day 4; sero-conversion was defined as a titre of serum neutralising antibodies to RV16 of at least 1:4 at 6 weeks.[2]

Diary Cards for Symptom Scores and Spirometry Recording. Symptom assessment was by daily diary cards, for 2 weeks before, during, and 6 weeks after infection. The daily cold score was summated from individual scores (sneezing, headache, malaise, chilliness, nasal discharge, nasal obstruction, sore throat, cough, fever) graded 0 (absent) to 3 (severe). The daily chest score was calculated from symptom scores (cough on waking; wheeze on waking; daytime cough; daytime wheeze; daytime shortness of breath; nocturnal cough, wheeze or shortness of breath), graded 0–3. The same diary cards recorded medication usage and home spirometry (microDL; MicroMedical) recording the best of three recordings of PEF and FEV1.[2]

Immunohistochemistry (IHC): EnVision-alkaline phosphatase technique (Dako Ltd, Cambridge, UK) was used to label CD45+ pan leukocyte/inflammatory cells, EG2+ eosinophils, neutrophil elastase+ neutrophils, tryptase+ mast cells and CD68+ monocytes/macrophages. EnVision peroxidase staining

©2014 American College of Chest Physicians. Reproduction of this article is prohibited without written permission from the American College of Chest Physicians. See online for more details. **DOI: 10.1378/chest.13-1567**
CHEST 2014; 145(6):1219-1229

method (Dako) was used to identify CD3+, CD4+, CD8+ T- and CD20+ B-lymphocytes as previously described but with minor modification.[3] Irrelevant mouse IgG_1 kappa antibody (MOPC21) was used to substitute for the primary layer as negative control for staining specificity of mouse monoclonal antibodies. The following panel of monoclonal mouse anti-human antibodies (Dako) was applied to tissue sections: anti-CD45 (M0701), neutrophil elastase (M0752), tryptase mast cell (M7052), CD3+ (M0835), CD4+ (M0716), CD8 (M0707) CD20 (M0755) and CD68 (M0876). Mouse anti-EG2 (EG2) was from Pharmacia & Upjohn Ltd, Milton Keynes, UK,

The immunostaining procedures for detecting the phenotypes of inflammatory cells were conducted by Techmate 'Horizon' automated immunostainer (LJL Biosystems Inc, USA).

Quantification

In histological slides, coded to avoid observer bias, areas of epithelium and subepithelium, excluding muscle and gland, were assessed using an Apple Macintosh computer and Image 1.5 software. Distinct phenotypes of inflammatory cells were counted using a Leitz Dialux 20 light microscope (Leitz Wetzlar, West Germany). Two to three bronchial biopsies for each subject were measured and counted in order to take account of within subject variability. The total epithelial and subepithelial areas of two or three biopsies from each bronchoscopy were measured and counted, respectively. Then the total counts were divided by total area to normalise the counts as the number of cells per unit. The data for bronchial biopsy cell counts were expressed as the number of cut cell profiles with a nucleus visible (i.e. positive cells) per mm^2 of the subepithelium and per 0.1 mm^2 epithelium. The coefficient of variation for repeat counts of cells immunopositive for sub-type markers of inflammatory cells by one observer this ranged between 5% and 6%.

Online supplements are not copyedited prior to posting.

©2014 American College of Chest Physicians. Reproduction of this article is prohibited without written permission from the American College of Chest Physicians. See online for more details. **DOI: 10.1378/chest.13-1567**
CHEST 2014; 145(6):1219-1229

Statistical analysis

Statistical analysis was performed using StatView (SAS Institute. Inc) and GraphPad Prism 4 (GraphPad Software, Inc) softwares.

One way ANOVA followed by the unpaired Student's t-test was used for the analyses of age, serum IgE, lung function and histamine PC_{20} data between groups. In respect of cell counts, these data were non-normally distributed and differences between groups were assessed first using the Kruskal-Wallis test, which, if significant, was followed by Wilcoxon matched pairs test within group between baseline and infection and Mann-Whitney U-test between groups at baseline and infection. The coefficient of variation ($CV = SD/mean \times 100$) was used to express the error of repeat counts. A P-value of < 0.05 was accepted as indicating a significant difference. Spearman's rank correlation was used as a test for correlations between the numbers of specific types of inflammatory cell and physiologic/clinical data. We wished to examine the relationships between virus load and the inflammatory response and believe that this is relevant in the non-asthmatic subjects also. Including the controls allowed us to examine these relationships in greater numbers of subjects and therefore avoid the possibility of missing significant correlations due to lack of power.

References

1. Sterk PJ, Fabbri LM, Quanjer PhH et al. Airway responsiveness. Standardized challenge testing with pharmacological, physical and sensitizing stimuli in adults. Report working party "Standardization of Lung Function Tests". European Community for Steel and Coal. Official statement of the European Respiratory Society. *Eur Respir J*. 1993;6 (Suppl 16):53-83.

2. Message SD, Laza-Stanca V, Mallia P et al. Rhinovirus-induced lower respiratory illness is increased in asthma and related to virus load and Th1/2 cytokine and IL-10 production. *Proc Natl Acad Sci U S A*. 2008;105:13562-13567.

3. O'Shaughnessy T, Ansari TW, Barnes NC et al. Inflammation in bronchial biopsies of subjects with chronic bronchitis: inverse relationship of $CD8^+$ T lymphocytes with FEV_1. *Am J Resp Crit Care Med*. 1997;155:852-857.

Online supplements are not copyedited prior to posting.

©2014 American College of Chest Physicians. Reproduction of this article is prohibited without written permission from the American College of Chest Physicians. See online for more details. **DOI: 10.1378/chest.13-1567**
CHEST 2014; 145(6):1219-1229
